# Comorbidity patterns and mortality in atrial fibrillation: a latent class analysis of the EURopean study of Older Subjects with Atrial Fibrillation (EUROSAF)

**DOI:** 10.1080/07853890.2025.2454330

**Published:** 2025-01-18

**Authors:** Huah Shin Ng, Richard Woodman, Nicola Veronese, Alberto Pilotto, Arduino A. Mangoni

**Affiliations:** a Department of Clinical Pharmacology, Flinders Medical Centre, Southern Adelaide Local Health Network, Adelaide, Australia; b Flinders Health and Medical Research Institute, College of Medicine and Public Health, Flinders University, Adelaide, Australia; c SA Pharmacy, SA Health, Adelaide, Australia; d Discipline of Biostatistics, College of Medicine and Public Health, Flinders University, Adelaide, Australia; e Geriatrics Unit, Department of Internal Medicine and Geriatrics, University of Palermo, Palermo, Italy; f Geriatrics Unit, Department of Geriatric Care, Neurology and Rehabilitation, Galliera Hospital, Genova, Italy; g Department of Interdisciplinary Medicine, “Aldo Moro” University of Bari, Bari, Italy

**Keywords:** Atrial fibrillation, mortality, older adults, comorbidities, anticoagulants, latent class analysis, phenotypes, frailty, multidimensional prognostic index.

## Abstract

**Background:**

Most older patients with atrial fibrillation (AF) have comorbidities. However, it is unclear whether specific comorbidity patterns are associated with adverse outcomes. We identified comorbidity patterns and their association with mortality in multimorbid older AF patients with different multidimensional frailty.

**Methods:**

Hospitalised adults aged ≥65 years with non-valvular AF were followed for 12 months in the multicentre EURopean study of Older Subjects with Atrial Fibrillation (EUROSAF). Demographic characteristics, coexisting medical conditions, use of medications including anticoagulants, and the Multidimensional Prognostic Index (MPI) were captured on discharge. We used latent class analysis (LCA) to identify comorbidity phenotypes and Cox regression to determine associations between identified phenotypes and 12-month mortality.

**Results:**

Amongst *n* = 2,019 AF patients (mean ± SD age 82.9 ± 7.5 years), a 3-class LCA solution was considered optimal for phenotyping. The model identified phenotype 1 (hypertensive, other circulatory conditions, metabolic diseases; 33%), phenotype 2 (digestive diseases, infection, injury, non-specific clinical and laboratory abnormalities; 26%), and phenotype 3 (heart failure, respiratory diseases; 41%). Overall, 512 patients (25%) died within 12 months. Compared to phenotype 1, after adjusting for age, sex, use of anticoagulants, cardiovascular medications, and proton pump inhibitors, and individual MPI domains, phenotype 3 had a significantly higher risk of mortality (adjusted hazard ratio = 1.27, 95% CI = 1.01 to 1.60). In contrast, the risk of mortality in phenotype 2 was not different to phenotype 1.

**Conclusion:**

We observed an association between comorbidity phenotypes identified using LCA and mortality in older AF patients. Further research is warranted to identify the mechanisms underpinning such associations.

## KEY MESSAGES

- This study showed a significant association between comorbidity phenotypes identified using latent class analysis and mortality in older patients with atrial fibrillation.
- Three distinct comorbidity phenotypes were identified, and the phenotype with a high prevalence of heart failure and respiratory diseases was associated with a 27% (95%CI 1% to 60%) higher risk of all-cause mortality.
- Further research is needed to investigate the molecular and pathophysiological mechanisms underpinning the interplay between atrial fibrillation, heart failure and respiratory diseases and identify new, personalized therapeutic avenues for this comorbidity pattern.

## Introduction

The prevalence of atrial fibrillation (AF), the most common chronic arrhythmia globally, increases progressively with age with epidemiological studies reporting figures between 9% and 17.8% in subjects aged ≥75 years [1–3]. AF patients have a significantly higher risk of heart failure, thromboembolic events, hospitalizations and death. Although there have been significant advances over the last two decades in diagnosis and treatment, including the availability of different types of anticoagulants [4,5], temporal trends and clinical experience have also highlighted additional challenges in the contemporary management of AF. Such challenges primarily include an increasing age-related number of people with AF worldwide and increasing patient complexity [6,7]. Such complexity is driven by the increasingly recognized role of frailty in influencing patient outcomes and treatment decision and the frequent coexistence of other disease states [7–9]. A recent study investigating 3.4 million people in the Clinical Practice Research Datalink in England showed a 30% increase in the incidence of AF between 1998 and 2017, with a crude incidence increasing with age [7]. The number of comorbidities also increased over time, from 2.58 (standard deviation, SD 1.83) in 1998 to 3.74 (SD 2.29) in 2017. Therefore, the study of the complex interplay between AF and other comorbid states, and the potential influencing role of specific medications (e.g. anticoagulants) and measures of frailty, is the focus of intense research [10,11].

Several studies have investigated the impact of comorbidity burden on clinical outcomes in patients with AF using conventional statistical methods. In these studies, comorbidity burden was assessed investigating specific comorbidities, calculating the total number of co-morbidities, or using established scoring systems such as the Charlson comorbidity index [12–15]. While providing valuable information, these studies have primarily assessed independent associations between individual comorbidities, or their total number, and clinical endpoints. This traditional variable-centred approach generates information that contrasts with various clustering methods that shift the focus of the analysis towards a patient-centred approach [16]. Specifically, such methods can be applied for identifying different AF subgroups, with each subgroup having a similar set of comorbidities. This is particularly useful in highly heterogeneous populations, e.g. AF patients with different comorbidities, frailty, and burden of polypharmacy [17–19], by facilitating targeted, cost-effective interventions to improve outcomes and quality of life, particularly in those groups with the highest risk.

One such clustering method, latent class analysis (LCA), is a ‘model-based’ method that relies on defined criteria (Akaike Information Criterion, AIC, and Bayesian Information Criterion, BIC) to indicate the optimal number of clusters, e.g. the number of different comorbidity patterns, in different populations [20–23]. The models and fit statistics using LCA have been demonstrated to perform consistently with high accuracy in studies with sample sizes larger than 500 [21], and the number of sample members in each class should not contain less than 5% of the sample [24]. However, there is limited previous work on how identifying different combinations of comorbidities and acute conditions in patients with AF may help improve their management and prognosis, particularly by identifying subgroups of individuals with higher risk and considering the patient in a more holistic manner than more traditional regression-based approaches that aggregate the risk from individual factors into a single overall risk score. The use of such overall risk scores generally performs poorly in practice [25] whilst precision medicine approaches that incorporate patient type into decision making have shown more promise [26,27]. For example, although the Multidimensional Prognostic Index (frailty assessment tool) is useful in triaging patients into 3 levels of overall risk, it again does not consider the patient phenotype which provides a patient-centred view for the treating clinician when deciding upon patient management and treatment. Therefore, in this study we used LCA to identify specific patterns of comorbidities in AF to answer the following three research questions: (i) what are different patterns of comorbidities and acute conditions in AF in a cohort of older patients characterized by substantial heterogeneity in the use of anticoagulants and other medications and measures of frailty?; (ii) what is the association between different patterns of comorbidities and mortality?; and (iii) what are the associations between the use of anticoagulants and mortality, and the association between frailty and mortality according to comorbidity patterns?

## Methods

### Data source and study population

We investigated AF patients participating in the EURopean study of Older Subjects with Atrial Fibrillation (EUROSAF). The study details have been described elsewhere [28–30]. Briefly, EUROSAF is a prospective observational study of a consecutive series of hospitalized AF patients ≥65 years across 24 geriatric centres in 12 European countries between 01/01/2016 and 31/12/2020. The inclusion criteria were hospital admission for an acute medical condition or relapse of a chronic condition, documented diagnosis of non-valvular AF, and willingness to participate and give informed consent. The exclusion criteria were inability to provide informed consent and death during hospital stay. Patients were followed for 12 months upon discharge from the hospital.

### Outcome measure

The primary outcome of the EUROSAF study was 12-month all-cause mortality [28–30].

### Variables collected

#### Comorbidities

We considered all short- or long-term coexisting conditions (comorbidities) together with the reasons for hospitalisation documented at the time of discharge in the analysis. These were extracted from medical records and coded using the International Classification of Diseases 10^th^ revision and categorised into 28 broad disease groupings/conditions: certain infectious and parasitic diseases, neoplasms, diseases of the blood and blood-forming organs, diseases of the nervous, respiratory, digestive, musculoskeletal, and genitourinary systems, mental and behavioural disorders, diseases of skin, eye, and ear, congenital malformations, non-specific clinical and laboratory abnormalities (e.g. general symptoms and signs not elsewhere classified or abnormal findings on examination of blood/urine/diagnostic imaging without diagnosis), injury, poisoning and certain other consequences of external causes, external causes of morbidity and mortality, and factors influencing health status and contact with health services. Within the endocrine and metabolic diseases, we separately collected data on diabetes mellitus and dyslipidaemia, and then grouped all other conditions as other endocrine/metabolic diseases. Within the diseases of the circulatory system, we separately collected data on rheumatic heart disease, hypertensive disease, ischemic heart disease, heart failure/cardiomyopathy/pulmonary hypertension, other forms of heart diseases, cerebrovascular diseases, deep vein thrombosis/pulmonary embolism, and then grouped all other conditions (i.e. disease of arteries, arterioles and capillaries, diseases of veins, lymphatic vessels and lymph nodes, and other and unspecified disorders of the circulatory system) as other circulatory diseases.

#### Medications

Anticoagulant use on discharge was categorized as follows: no anticoagulant use, use of vitamin K antagonists (e.g. warfarin, dicoumarol, phenindione, and acenocoumarol), or use of direct oral anticoagulants (DOACs; e.g. rivaroxaban, apixaban, edoxaban, and dabigatran). Information was collected on thromboembolic risk assessed using the CHA2DS2-VASC score (congestive heart failure, hypertension, age category, diabetes, stroke, vascular disease, sex) and bleeding risk assessed using the HAS-BLED score (hypertension, abnormal liver or renal function, stroke, bleeding, labile international normalised ratio, old age, drugs or alcohol).

We also captured information regarding seven commonly used drug classes other than anticoagulants, according to the World Health Organisation Anatomical Therapeutic Chemical classification system: selected cardiovascular medications including antiplatelet agents (B01AC), cardiac therapy (C01), diuretics (C03), beta-blocking agents (C07), agents acting on the renin-angiotensin system (C09), lipid-modifying agents (statins, C10AA), and proton pump inhibitors (A02BC).

#### Multidimensional Prognostic Index

We collected data on the eight individual domains of the Multidimensional Prognostic Index (MPI), a validated prognostic index derived from the Comprehensive Geriatric Assessment which includes robust measures of frailty [31–33], on discharge: 1) activities of daily living index; 2) instrumental activities of daily living; 3) short portable mental status questionnaire; 4) mini nutritional assessment short form; 5) Exton-Smith scale; 6) number of medications taken; 7) cumulative illness rating scale-comorbidity index score; and 8) cohabitation status (Supplement Material 1). A value (0 = no problem, 0.5 = minor problems or 1 = major problems) was assigned to each domain based on the corresponding risk and a final MPI score was calculated by dividing the sum of the values from each domain by eight. MPI was then categorised into 3 classes based on the final MPI score: MPI-1 (robust with MPI score 0.0-0.33), MPI-2 (pre-frailty with MPI score 0.34-0.66), and MPI-3 (frailty with MPI score 0.67-1.00) [28–30]. All data collected were anonymised and de-identified for analysis.

### Statistical analysis

The cohort characteristics were presented using descriptive analysis including frequency (percentages) for categorical variables and mean (SD) for continuous variables. We explored comorbidity patterns based on 28 broad disease groupings/conditions described above using LCA and trialled models containing between two and six classes. Model selection was based on BIC statistics and model parsimony [20]. Based on the lowest BIC, a three-class model was selected. Classes were subjectively labelled by first calculating the observed-to-expected (O/E) ratio of each comorbidity condition in each class and then identifying the most discriminating comorbidity conditions. The expected prevalence of comorbidity was based on the overall prevalence of each comorbidity within the total cohort. Conditions with an O/E ratio >1.7 were considered as the most discriminating comorbidities and used for the labelling of each phenotype (Supplementary Table 1).

We examined the association between comorbidity patterns (phenotypes) and 12-month all-cause mortality using multivariable Cox regression models. The study end date was the earlier of death or loss to follow-up or at the end of the 12-month follow-up period. Subjects were followed until the date of death (time to event of interest) or censored at the date they were lost to follow up or at the end of the 12-month follow-up period, whichever date come first. Analyses included four levels of adjustment: model 1= adjustment for sex and age alone; model 2 = model 1 + adjustment for anticoagulant treatment; model 3 = model 2+ adjustment for use of selected cardiovascular medications and proton pump inhibitors; and model 4 = model 3 + adjustment for individual domains of the MPI. Results were reported as adjusted hazard ratios (aHR) with 95% confidence interval. The assumptions of proportional hazards were examined by including an interaction term between covariates and log (follow-up). We also examined separately the association between the use of anticoagulant therapy and mortality for each of the comorbidity phenotypes (model 4 adjustment), and the association between MPI classes (frailty) and mortality for each of the comorbidity phenotypes (model 3 adjustment). All statistical analyses were performed using SAS software version 9.4 (Cary, NC, USA). LCA was conducted with StepMix using Python version 3.9.13.

### Ethics approval

The study was approved by the ethics committee of each participating centre (ethics registration details of the leading centre, Ente Ospedaliero Genova, Italy: 162REG2016). The study protocol was registered in ClinicalTrials.gov (NCT02973984). The study adheres to the Declaration of Helsinki and written informed consent was given by participants.

## Results

### Study population and phenotypes

**Figure 1.**
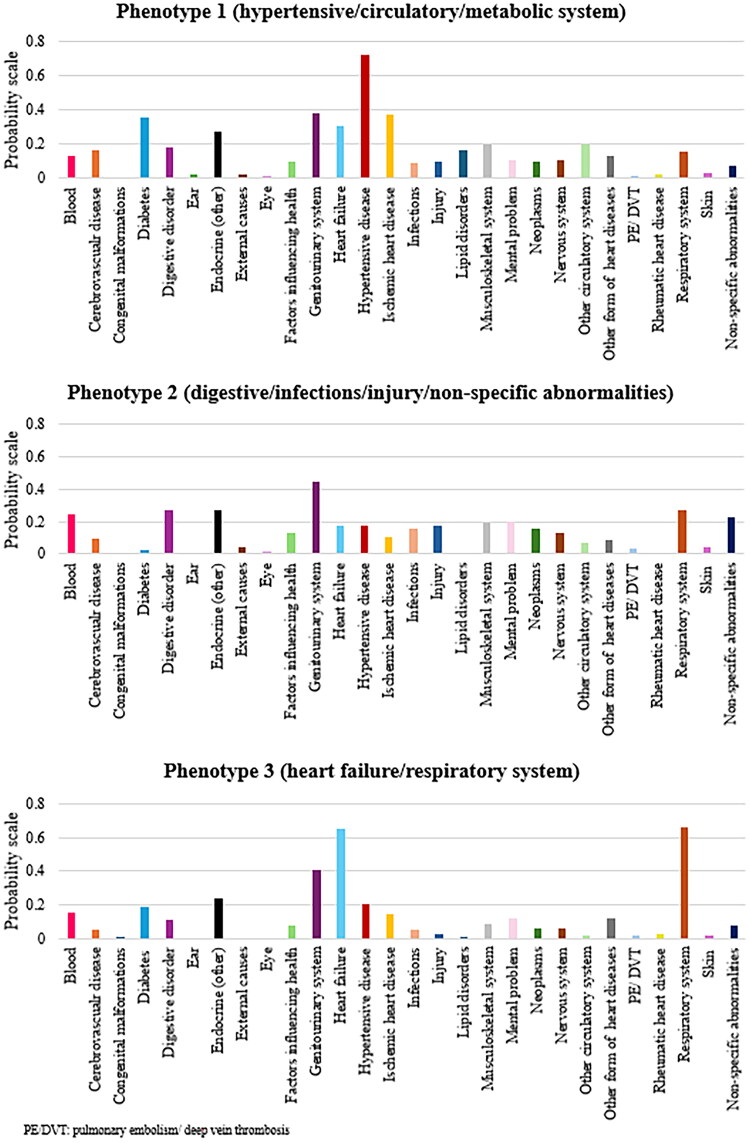
Three-class model of comorbidities identified by latent class analysis.

The study population included *n* = 2,019 older hospitalised adults with AF (mean ± SD age 82.9 ± 7.5 years, 57% female; Table 1). Based on the O/E ratio for comorbidities, the three LCA identified comorbidity patterns were labelled as phenotype 1 (hypertensive, circulatory, metabolic system diseases; *n* = 672, 33%), phenotype 2 (digestive diseases, infection, injury, non-specific clinical and laboratory abnormalities; *n* = 515, 26%), and phenotype 3 (heart failure, respiratory diseases; *n* = 832, 41%; Figure 1 and Supplementary Table 1). A higher proportion of individuals belonging to phenotype 3 were treated with anticoagulants compared to the phenotypes 2 and 1 (63% vs. 54 and 59% respectively; Table 1). Phenotype 1 also had a higher mean CHA2DS2-VASC score than the other two phenotypes. By contrast, there were no significant differences in the risk of major bleeding across the three phenotypes. The use of selected cardiovascular medications, e.g. drugs acting on the renin-angiotensin system, and lipid-modifying agents (statins) was significantly higher in phenotype 1 whereas the use of diuretics was significantly higher in phenotype 3, likely reflecting the variation in individual conditions across phenotypes. Furthermore, phenotype 1 was associated with a significantly higher level of independence and functional and cognitive status, and a lower prevalence of pressure sores when compared to phenotype 2 and phenotype 3.

**Table 1.** Characteristics of the study cohort and individual patient phenotypes.

	Total cohort (*n* = 2019)	Phenotype 1 (*n* = 672)	Phenotype 2 (*n* = 515)	Phenotype 3 (*n* = 832)	P-value^a^
	All study population	Hypertensive/ circulatory/ metabolic system	Digestive/ infections/ injury/non-specific abnormalities	Heart failure/ respiratory system	
Female sex	1144 (57%)	394 (59%)	291 (57%)	459 (55%)	0.4296
Age in years, mean (SD)	82.9 (7.5)	81.0 (7.3)	83.6 (7.4)	84.2 (7.4)	<0.0001
Anticoagulants					
No anticoagulant	820 (41%)	278 (41%)	237 (46%)	305 (37%)	0.0001
Any anticoagulants	1199 (59%)	394 (59%)	278 (54%)	527 (63%)	
Vitamin K antagonist	450 (22%)	142 (21%)	85 (17%)	223 (27%)	
Direct oral anticoagulants	749 (37%)	252 (38%)	193 (37%)	304 (36%)	
CHA2DS2-VASC, mean (SD)	4.9 (1.5)	5.2 (1.4)	4.6 (1.5)	4.8 (1.4)	<0.0001
HAS-BLED, mean (SD)	2.8 (1.1)	2.8 (1.1)	2.8 (1.2)	2.7 (1.1)	0.3536
Other medications					
Antiplatelet agents	366 (18%)	138 (21%)	86 (17%)	142 (17%)	0.1378
Cardiac therapy	587 (29%)	204 (30%)	141 (27%)	242 (29%)	0.5341
Diuretics	1356 (67%)	411 (61%)	273 (53%)	672 (81%)	<0.0001
Beta-blockers	1364 (68%)	454 (68%)	332 (64%)	578 (69%)	0.1624
Agents acting on RAS	971 (48%)	389 (58%)	196 (38%)	386 (46%)	<0.0001
Statins	598 (30%)	267 (40%)	119 (23%)	212 (25%)	<0.0001
Proton pump inhibitors	1162 (58%)	389 (58%)	295 (57%)	478 (57%)	0.9755
MPI domains					
ADL, mean (SD)	3.7 (2.2)	4.2 (1.9)	3.3 (2.3)	3.6 (2.2)	<0.0001
IADL, mean (SD)	3.7 (2.8)	4.5 (2.7)	3.2 (2.8)	3.3 (2.8)	<0.0001
SPMSQ, mean (SD)	2.9 (2.9)	2.2 (2.5)	3.2 (3.2)	3.2 (3.0)	<0.0001
MNA, mean (SD)	9.3 (3.1)	9.8 (3.0)	8.5 (3.0)	9.5 (3.0)	<0.0001
ESS, mean (SD)	15.7 (3.3)	16.5 (3.1)	15.1 (3.4)	15.5 (3.4)	<0.0001
Number of medications, mean (SD)	7.7 (3.3)	7.4 (3.5)	8.0 (3.2)	7.7 (3.2)	0.0188
CIRS-CI, mean (SD)	4.1 (2.3)	3.8 (2.2)	4.6 (2.2)	4.0 (2.3)	<0.0001
Living alone, n (%)	564 (28%)	209 (31%)	163 (32%)	192 (23%)	<0.0001
MPI score, mean (SD)	0.49 (0.21)	0.44 (0.20)	0.54 (0.20)	0.50 (0.20)	<0.0001
MPI class					
MPI-1 (robust)	568 (28%)	250 (37%)	102 (20%)	216 (26%)	<0.0001
MPI-2 (pre-frailty)	949 (47%)	306 (46%)	244 (47%)	399 (48%)	
MPI-3 (frailty)	502 (25%)	116 (17%)	169 (33%)	217 (26%)	

Legend: SD, standard deviation; CHA2DS2-VASC, congestive heart failure, hypertension, age category, diabetes, stroke, vascular disease, sex category; HAS-BLED, hypertension, abnormal liver or renal function, stroke, bleeding, labile INR, old age, drugs or alcohol; RAS, renin angiotensin system; MPI, multidimensional prognostic index; ADL, activities of daily living index; IADL, instrumental activities of daily living; SPMSQ, short portable mental status questionnaire; MNA, mini nutritional assessment short form; ESS, Exton-Smith scale; CIRS-CI, cumulative illness rating scale-comorbidity index score.

^a^Comparisons between 3 phenotypes were performed using chi-square test for categorical variables, and ANOVA test for continuous variables.

### Mortality

**Figure 2.**
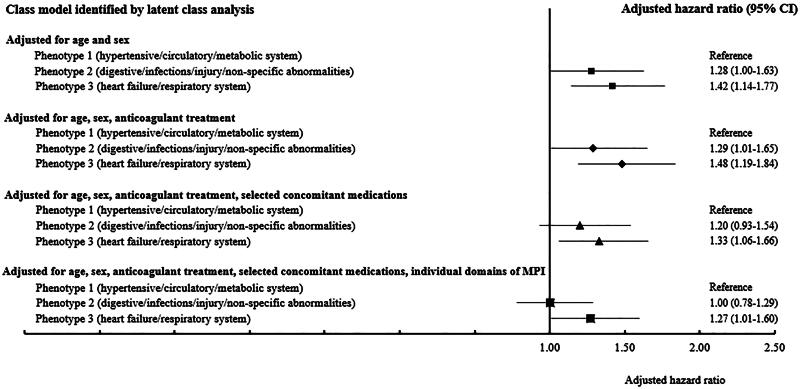
Association between individual phenotypes and all-cause mortality adjusted for selected covariates. Legend: CI, confidence interval; MPI, multidimensional prognostic index. Number of people who died: Phenotype 1 (n = 132/672; 20%), Phenotype 2 (n = 135/515; 26%), Phenotype 3 (n = 245/832; 29%)

One quarter (*n* = 512/2019, 25%) of participants died within 12 months of follow-up, with a higher proportion of deaths in phenotype 3 (*n* = 245/587, 29%; Table 2). The fully adjusted risk of mortality (model 4) was also higher in phenotype 3 when compared to phenotype 1 (aHR = 1.27, 95%CI = 1.01-1.60). By contrast, there was no significant difference in mortality risk between phenotype 2 and phenotype 1 (aHR = 1.00, 95%CI = 0.78-1.29; Figure 2 & Supplementary Figure 1).

**Table 2.** Association between anticoagulant therapy, MPI category and mortality by phenotype.

	Total cohort (*n* = 2019)	Phenotype 1 (*n* = 672)	Phenotype 2 (*n* = 515)	Phenotype 3 (*n* = 832)
	All study population	Hypertensive/ circulatory/ metabolic system	Digestive/ infections/ injury/non-specific abnormalities	Heart failure/ respiratory system
(a) Anticoagulant therapy				
Number of people who died (%)				
No anticoagulant	279/820 (34%)	78/278 (28%)	78/237 (33%)	123/305 (40%)
Vitamin K antagonist	109/450 (24%)	28/142 (20%)	21/85 (25%)	60/223 (27%)
Direct oral anticoagulants	124/749 (17%)	26/252 (10%)	36/193 (19%)	62/304 (20%)
Total cohort	512/2019 (25%)	132/672 (20%)	135/515 (26%)	245/832 (29%)
Hazard ratios^a^ (95% confidence interval) for 12-month mortality				
No anticoagulant	1.00 (Ref)	1.00 (Ref)	1.00 (Ref)	1.00 (Ref)
Vitamin K antagonist	0.79 (0.62-1.01)	1.00 (0.61-1.61)	0.81 (0.48-1.36)	0.66 (0.47-0.93)
Direct oral anticoagulants	0.48 (0.38-0.60)	0.46 (0.28-0.75)	0.51 (0.33-0.78)	0.45 (0.32-0.62)
(b) Multidimensional prognostic index (MPI) category				
Number of people who died (%)				
MPI-1 (robust)	78/568 (14%)	20/250 (8%)	13/102 (13%)	45/216 (21%)
MPI-2 (pre-frailty)	216/949 (23%)	65/306 (21%)	52/244 (21%)	99/399 (25%)
MPI-3 (frailty)	218/502 (43%)	47/116 (41%)	70/169 (41%)	101/217 (47%)
Total cohort	512/2019 (25%)	132/672 (20%)	135/515 (26%)	245/832 (29%)
Hazard ratios^b^ (95% confidence interval) for 12-month mortality				
MPI-1 (robust)	1.00 (Ref)	1.00 (Ref)	1.00 (Ref)	1.00 (Ref)
MPI-2 (pre-frailty)	1.60 (1.22-2.09)	2.45 (1.44-4.18)	2.03 (1.04-3.96)	1.14 (0.79-1.64)
MPI-3 (frailty)	3.18 (2.40-4.22)	4.79 (2.69-8.52)	4.16 (2.14-8.10)	2.28 (1.54-3.37)

Legend: MPI, multidimensional prognostic index; Ref, reference category.

^a^Adjusted for age, sex, selected cardiovascular medications and proton pump inhibitors, and individual domains of the multidimensional prognostic index.

^b^Adjusted for age, sex, selected cardiovascular medications and proton pump inhibitors, and anticoagulant therapy.

The use of DOACs was associated with a lower hazard of mortality for each of the three phenotypes (aHR range from 0.45 to 0.51) as well as overall (aHR = 0.48, 95%CI 0.38-0.60) whereas the use of vitamin K antagonists was associated with lower mortality only in phenotype 3 (aHR = 0.66, 95%CI 0.47-0.93; Table 2).

Frailty assessed by MPI was associated with a higher hazard of mortality for each of the three phenotypes (aHR range from 2.28 to 4.79) as well as overall (aHR = 3.18, 95%CI 2.40-4.22; Table 2).

## Discussion

In this study, the use of LCA, an increasingly popular clustering method in medical research, led to the identification of three distinct comorbidity phenotypes in older hospitalised patients with AF. Phenotype 3 (heart failure, respiratory diseases) was associated with a significantly higher (27%, 95%CI 1% to 60%) risk of 12-month all-cause mortality when compared to phenotype 1 (hypertensive, circulatory, metabolic system diseases). In addition, the risk of mortality in phenotype 2 (digestive diseases, infection, injury, non-specific clinical and laboratory abnormalities) was virtually identical to that in phenotype 1. Notably, the increased risk of mortality observed in phenotype 3 was independent of other important factors that have been shown to be associated with this endpoint in previous studies, including age [34], sex [35,36], the use of anticoagulants [30,37], and measures of frailty [8, 38]. We also found that compared to no anticoagulant use, the use of anticoagulants particularly DOACs was associated with a similar and significantly lower risk of mortality in all three phenotypes, whereas increased frailty was associated with a higher risk of mortality in all three phenotypes.

Other studies have used different clustering techniques such as hierarchical clustering method and K-prototype to examine the patterns of comorbidities in AF and their association with mortality [39–46]. For example, Proietti et al. used a hierarchical cluster analysis (Ward’s method and Squared Euclidean Distance) that included 22 clinical variables in the European EORP-AF Registry (median age = 71 years, interquartile range = 63-77) and identified three clusters [46]. Compared to cluster 2 (younger people with few comorbidities), cluster 3 (prevalent cardiovascular risk factors and comorbidities) was associated with significantly higher mortality [46]. Saito et al. used the K-prototype clustering method in two observational Japanese registries (SAKURA AF, mean ± SD age 72 ± 9 years; and RAFFINE, mean ± SD age 73 ± 10 years) that included 14 clinical variables and identified five clusters [39]. Compared to cluster 1 (youngest age, highest body mass index, lowest rate of ischemic heart disease and B-type natriuretic peptide), a significant increase in the risk of mortality was observed in the other clusters, particularly cluster 4 (oldest, female, lowest body mass index, hypertension, heart failure, anaemia, chronic kidney disease) and cluster 5 (older, male, long-standing AF, diabetes, hypertension, heart failure, ischaemic heart disease) [39]. While different cluster groupings (ranging from three to six clusters) were identified in these previous studies, at least partly due to heterogeneity of the study population and differences in the types of clinical, laboratory and comorbidity variables included in the analyses, two phenotypes appeared consistent across studies [39–46]. A first phenotype comprised of younger patients with a low burden of comorbidity, and a second phenotype composed of people with a high burden cardiovascular comorbidities and associated with worse outcomes. While providing useful information regarding the association between specific clusters and all-cause mortality, these studies assessed relatively younger cohorts with lower rates of non-cardiovascular conditions and medication use [39–46]. Furthermore, the confounding role of frailty was not assessed.

A recent study by Romiti et al. analysing the prospective and international GLORIA-AF Registry (mean ± SD age 70 ± 10 years), used LCA and identified six comorbidity phenotypes [47]. A higher risk of mortality was observed in four phenotypes (atherosclerotic, thromboembolic, cardiometabolic, high complexity, i.e. those with high burden of both cardiovascular and non-cardiovascular conditions) compared to the low complexity class (i.e. low prevalence of most comorbid conditions), while a lower risk of mortality was found in one phenotype (the cardiovascular risk factors class with youngest age and a high prevalence of hypertension, obesity, and hyperlipidaemia). Our findings are at least partially in line with the results of this study, indicating a significantly higher risk of mortality in AF patients with concomitant cardiovascular and non-cardiovascular conditions (phenotype 3; heart failure, respiratory diseases) when compared to phenotype 1, which also included hypertension and metabolic conditions such as hyperlipidaemia. Our study further advances the knowledge in the field as we were not only able to assess patterns of comorbidities but also to account for individual domains of the MPI, which includes robust measures of frailty. The MPI was a critical component of the EUROSAF study which primarily focused on complex multimorbid AF patients with different degrees of frailty status and polypharmacy [31–33]. The necessity to conduct further research in this group has recently been highlighted in an international consensus document endorsed by professional arrhythmias societies across different continents [48]. Specifically, the document acknowledges an important knowledge gap regarding how the management of AF can affect disability and frailty development.

Comorbidities including heart failure and respiratory diseases (e.g. chronic obstructive pulmonary disease) are present in up to about one-quarter and two-third of patients with AF, respectively [49–51]. The results of our study which showed a higher risk of mortality especially among those with concomitant cardiovascular and non-cardiovascular conditions (phenotype 3; heart failure, respiratory diseases) underscore the importance of managing these comorbidities in line with the Atrial Fibrillation Better Care pathway to provide an integrated and holistic approach which also applies to frail AF patients [48,52]. Adherence to the Atrial Fibrillation Better Care pathway has been demonstrated to be associated with reduced risk of mortality including in patients with multimorbidity [53]. Lifestyle modifications such as increased exercise/physical activity, smoking cessation and weight loss have been shown to improve the clinical outcomes in patients with AF and other diseases such as heart failure and chronic obstructive pulmonary disease [49,50]. Such interventions can be further enhanced through continuous patient education for risk reduction and early prevention. Our study also identified a similar risk of death between phenotype 1 (hypertensive, other circulatory conditions and metabolic diseases) and phenotype 2 (digestive diseases, infection, injury, non-specific clinical and laboratory abnormalities). This observation suggests that selected infections and injuries leading to acute hospitalisation, abnormal laboratory findings (e.g. lipids, glucose, and blood cells), and non-specific signs and symptoms (e.g. disorientation, dizziness and giddiness, and abnormalities of heart beat/blood pressure) not elsewhere classified in the International Classification of Diseases classification system may represent important health risk factors for 12-month mortality that warrant further investigation for risk management.

The use of anticoagulant particularly DOACs was associated with reduced mortality in all three comorbidity phenotypes. Our findings are consistent with other studies which showed beneficial effects of DOACs in older adults, including frail AF patients [54,55], and those with different types of comorbidities [37]. Phenotype 3 (heart failure, respiratory diseases) was associated with a significantly higher risk of mortality despite a higher proportion of individuals treated with anticoagulants compared to the other two phenotypes. This suggests that other factors such as significant comorbidities and frailty may contribute to a higher mortality risk in this patient population. Whilst frailty measured by the MPI was associated with a higher risk of death across the three phenotypes, the magnitude of effects varied by comorbidity patterns. It is possible that the severity of comorbidity may come into play whereby frailty may have a greater impact on death among people with less serious/severe types of health conditions (e.g. hypertension in phenotype 1 or digestive disorder in phenotype 2 versus more serious conditions such as heart failure in phenotype 3) that warrant further investigation in future studies.

Strengths of our study included the multicentre prospective design and the availability of a wide range of data capturing information on multiple comorbid states, use of anticoagulants, cardiovascular medications and proton pump inhibitors, and robust measures of frailty. An important limitation was the lack of information regarding the cause of death, e.g. cardiovascular vs. non-cardiovascular. Further, patients included in our study were limited to hospitalised older adults, and therefore, the generalisability of our findings to other settings remain to be explored. Multidimensional frailty assessment was determined at hospital discharge and therefore people who died during hospitalisation that may have also been more frail were excluded from the analysis, possibly introducing selection bias. While we considered all diagnosis codes including reasons for hospitalisation and coexisting conditions that may be short- or long-term recorded at the time of discharge, further studies are needed to examine longitudinal changes in comorbidities over time and their impact on health outcomes. We also cannot rule out the contribution of other unaccounted confounders. For example, while we adjusted for several important patient characteristics in the analysis, we were not able to account for information not captured in the datasets such as clinical and situational factors that may have influenced the choices of anticoagulant and drug treatment for medical conditions in the acute settings (introducing confounding by clinical indication) and other lifestyle factors (e.g. smoking status, alcohol intake). It is also possible that a larger dataset may offer additional phenotypes that differentiate 3 classes into additional subtypes that are also clinically relevant, and which may provide a more nuanced picture of phenotypes and their association with mortality.

## Conclusions

In conclusion, the use of LCA allowed the identification of three separate comorbidity phenotypes in older AF patients participating in the multicentre EUROSAF study. Compared to phenotype 1 (hypertensive, other circulatory conditions, metabolic diseases), the risk of 12-month all-cause mortality was significantly higher in phenotype 3 (heart failure, respiratory diseases) but not phenotype 2 (digestive diseases, infection, injury, non-specific clinical and laboratory abnormalities). The increased mortality in phenotype 3 was independent of age, sex, use of anticoagulants, cardiovascular medications and proton pump inhibitors, and individual MPI domains. Our results suggest the need for further research to investigate the molecular and pathophysiological mechanisms underpinning the interplay between AF, heart failure and respiratory diseases and identify new, personalized therapeutic avenues for this comorbidity pattern.

## Supplementary Material

Supplemental material .docx

## Data Availability

The de-identified data can be accessed upon reasonable request to the EUROSAF Study Investigators (Nicola Veronese, Alberto Pilotto).
