# Supplementary material for: Comorbidity patterns and mortality in atrial fibrillation: a latent class analysis of the EURopean study of Older Subjects with Atrial Fibrillation (EUROSAF)
Source: Ann Med. 2025 Jan 18;57(1):2454330. doi: 10.1080/07853890.2025.2454330 (PMC11749148; doi:10.1080/07853890.2025.2454330)
Supplement: Supplemental material .docx [file IANN_A_2454330_SM7376.docx]

**Supplement Material 1.** Computation of MPI score and classes

Individual MPI domains:

1) activities of daily living index (ADL): measure level of dependence/independence based on six daily personal care activities such as toileting, feeding, bathing^e1^

2) instrumental activities of daily living (IADL): assess functioning in the community based on eight more complex activities such as managing finances, preparing meals, taking medicines^e2^

3) short portable mental status questionnaire (SPMSQ): assess cognitive functioning with 10-item questionnaire such as orientation to time and place^e3^

4) mini nutritional assessment short form (MNA): evaluate nutritional status including changes in food take, recent weight loss^e4^

5) Exton-Smith scale (ESS): measure risk of pressure sores with five-item questionnaire including assessment of physical and mental condition, mobility, and incontinence)^e5^

6) number of medications taken.

7) cumulative illness rating scale-comorbidity index score (CIRS-CI): assess the severity of conditions across 13 systems with moderate or severe levels^e6^

8) cohabitation status (e.g., living alone or with family or living in an institution)

Score in each MPI domain:

| **MPI domain** | **Score in each domain** | | |
| --- | --- | --- | --- |
|  | **No problem (value= 0)** | **Minor problems (value= 0.5)** | **Major problems (value= 1)** |
| ADL | 6-5 | 4-3 | 2-0 |
| IADL | 8-6 | 5-4 | 3-0 |
| SPMSQ | 0-3 | 4-7 | 8-10 |
| MNA | 12-14 | 8-11 | 0-7 |
| ESS | 16-20 | 10-15 | 5-9 |
| Number of medications | 0-3 | 4-6 | ≥7 |
| CIRS-CI | 0 | 1-2 | ≥3 |
| Cohabitation status | Family | Nursing home | Alone |

Legend: MPI, multidimensional prognostic index; ADL, activities of daily living index; IADL, instrumental activities of daily living; SPMSQ, short portable mental status questionnaire; MNA, mini nutritional assessment short form; ESS, Exton-Smith scale; CIRS-CI, cumulative illness rating scale-comorbidity index score.

Calculation of total MPI score:

Total MPI score is calculated by dividing the value from each domain by eight.

Categorisation of MPI classes based on total MPI score:

1. MPI-1: robust (with MPI score from 0.00 to 0.33)
2. MPI-2: pre-frailty (with MPI score from 0.34 to 0.66)
3. MPI-3: frailty (with MPI score from 0.67 to 1.00)

References:

e1. Wallace M, Shelkey M, Hartford Institute for Geriatric N. Katz Index of Independence in Activities of Daily Living (ADL). Urol Nurs. 2007;27(1):93-4.

e2. Lawton MP, Brody EM. Assessment of older people: self-maintaining and instrumental activities of daily living. Gerontologist. 1969;9(3):179-86.

e3. Pfeiffer E. A short portable mental status questionnaire for the assessment of organic brain deficit in elderly patients. J Am Geriatr Soc. 1975;23(10):433-41.

e4. Kaiser MJ, Bauer JM, Ramsch C, Uter W, Guigoz Y, Cederholm T, et al. Validation of the Mini Nutritional Assessment short-form (MNA-SF): a practical tool for identification of nutritional status. J Nutr Health Aging. 2009;13(9):782-8.

e5. Bliss MR, McLaren R, Exton-Smith AN. Mattresses for preventing pressure sores in geriatric patients. Mon Bull Minist Health Public Health Lab Serv. 1966;25:238-68.

e6. Salvi F, Miller MD, Grilli A, Giorgi R, Towers AL, Morichi V, et al. A manual of guidelines to score the modified cumulative illness rating scale and its validation in acute hospitalized elderly patients. J Am Geriatr Soc. 2008;56(10):1926-31.

**Supplementary Table 1.** Observed/expected ratio per comorbidity by latent class analysis.

| **O/E ratio per comorbidity^a^** | **Latent classes** | | |
| --- | --- | --- | --- |
|  | **Phenotype 1 (Hypertensive/ circulatory/ metabolic system) (n=672)** | **Phenotype 2 (Digestive/ infections/injury/ non-specific abnormalities)**  **(n=515)** | **Phenotype 3 (Heart failure/respiratory system)**  **(n=832)** |
| Blood and blood forming organ | 0.78 | 1.59 | 0.81 |
| Cerebrovascular disease | **1.79** | 0.91 | 0.42 |
| Diabetes mellitus | **1.87** | 0.02 | 0.91 |
| Digestive disorder | 1.05 | **1.70** | 0.52 |
| Endocrine (other) | 1.07 | 1.09 | 0.88 |
| Factors influencing health status | 1.00 | 1.34 | 0.79 |
| Genitourinary system | 0.90 | 1.17 | 0.98 |
| Heart failure | 0.71 | 0.23 | **1.71** |
| Hypertensive disease | **2.17** | 0.32 | 0.48 |
| Ischemic heart disease | **1.86** | 0.47 | 0.64 |
| Infectious and parasitic diseases | 0.85 | **2.13** | 0.43 |
| Injury, poisoning and external causes | 1.06 | **2.18** | 0.22 |
| Lipid disorders | **3.00** | 0.00 | 0.00 |
| Musculoskeletal system | 1.38 | 1.30 | 0.51 |
| Mental and behavioural problem | 0.68 | 1.64 | 0.86 |
| Neoplasms | 0.63 | 1.16 | 0.39 |
| Nervous system | 1.08 | 1.49 | 0.64 |
| Other circulatory system disorder | **2.21** | 0.80 | 0.14 |
| Other form of heart diseases | 1.25 | 0.68 | 1.00 |
| Respiratory system | 0.30 | 0.58 | **1.82** |
| Non-specific clinical/laboratory abnormalities | 0.55 | **2.27** | 0.58 |

^a^O/E ratios were obtained by dividing the observed prevalence from latent class analysis by the expected prevalence from the total sample.

O/E ratios for seven conditions with prevalence <5% (congenital malformation, diseases of ear, external causes of morbidity/mortality, eye disorder, pulmonary embolism/deep vein thrombosis, rheumatic heart disease, and skin diseases) were not reported.

In each class, comorbidities with O/E ratio >1.7 are highlighted in bold, representing the most discriminating conditions used for the labelling of each phenotype.

**Supplementary Figure 1.** Survival curves for all-cause mortality by phenotypes adjusted for selected covariates

| 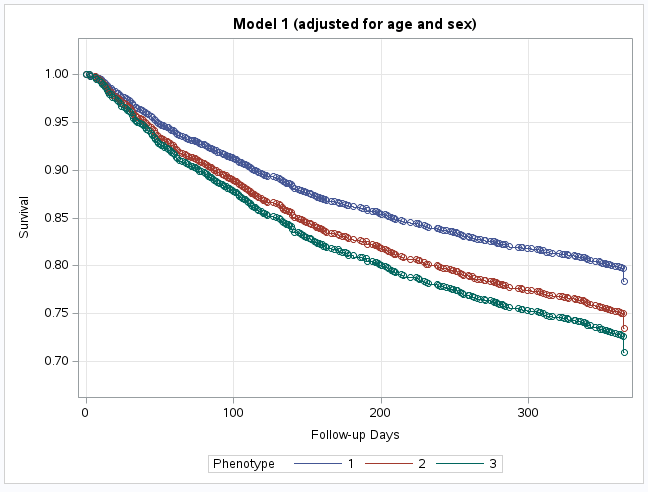 |
| --- |
| 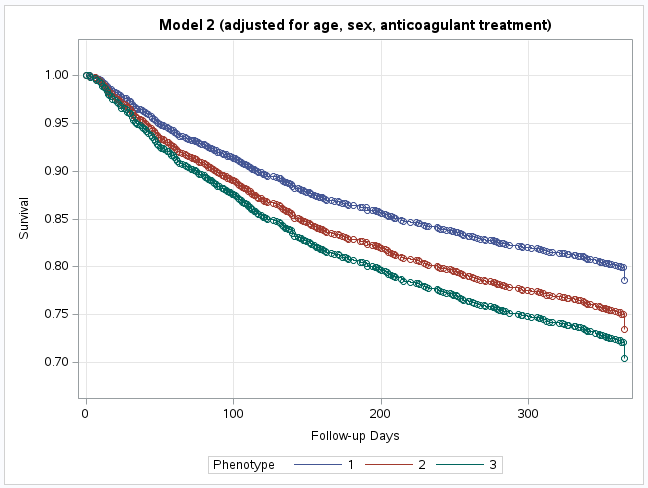 |

Phenotype 1 (Hypertensive/circulatory/metabolic system)

Phenotype 2 (Digestive/infections/injury/non-specific abnormalities)

Phenotype 3 (Heart failure/respiratory system)

| 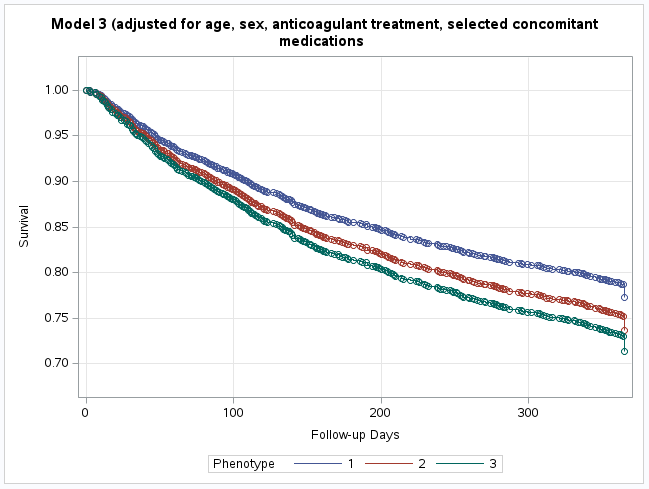 |
| --- |
| 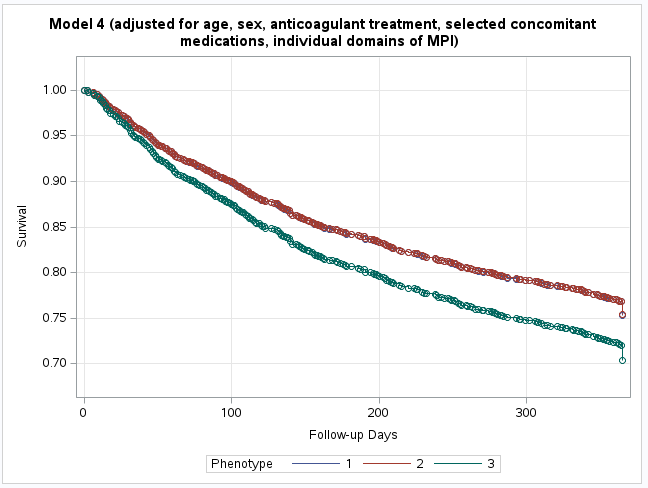 |

Model 4: phenotypes 1 and 2 have essentially the same risk of mortality after adjusting for their frailty

Phenotype 1 (Hypertensive/circulatory/metabolic system)

Phenotype 2 (Digestive/infections/injury/non-specific abnormalities)

Phenotype 3 (Heart failure/respiratory system)
